# Lipidomics Characterization of the Microbiome in People with Diabetic Foot Infection Using MALDI-TOF MS

**DOI:** 10.1021/acs.analchem.3c03071

**Published:** 2023-10-25

**Authors:** Justyna Walczak-Skierska, Fernanda Monedeiro, Ewelina Maślak, Michał Złoch

**Affiliations:** † Centre for Modern Interdisciplinary Technologies, Nicolaus Copernicus University in Toruń, Wileńska 4 Str., 87-100 Toruń, Poland; ‡ Chair of Environmental Chemistry and Bioanalytics, Faculty of Chemistry, Nicolaus a Copernicus University in Toruń, Gagarina 7 Str., 87-100 Toruń, Poland

## Abstract

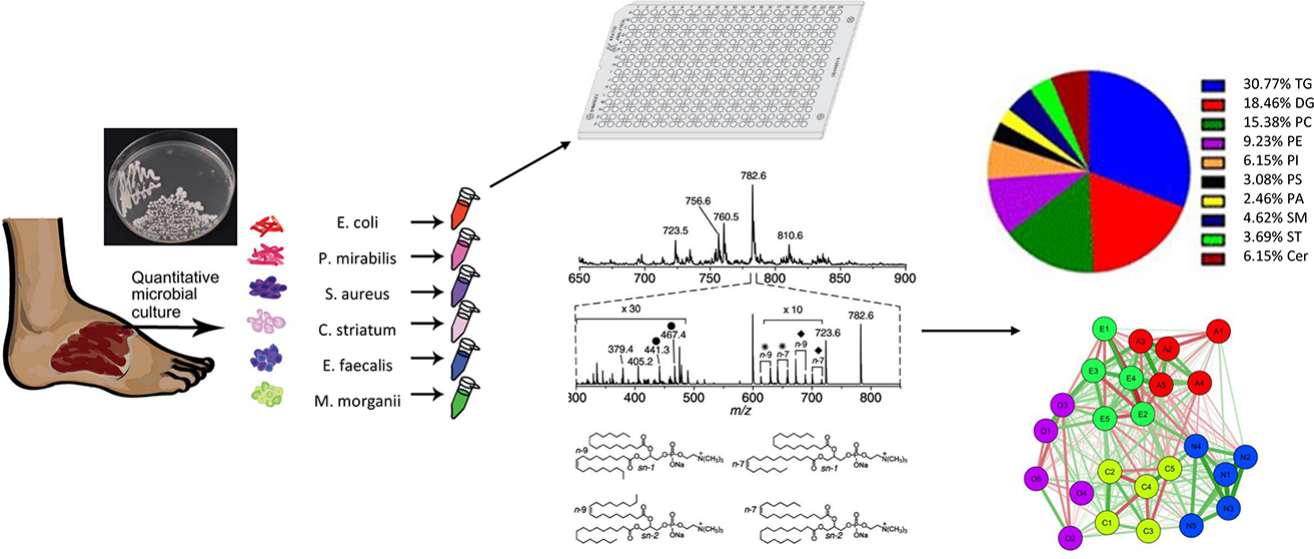

Lipidomic profiling has emerged as a powerful tool for the comprehensive characterization of bacterial species, particularly in the context of clinical diagnostics. Utilizing matrix-assisted laser desorption/ionization time-of-flight mass spectrometry (MALDI-TOF MS), this study aims to elucidate the lipidomic landscapes of bacterial strains isolated from diabetic foot infections (DFI). Our analysis successfully identified a diverse array of lipids in the cellular membranes of both Gram-positive and Gram-negative bacteria, revealing a total of 108 unique fatty acid combinations. Specifically, we identified 26 LPG, 33 LPE, 43 PE, 114 PG, 89 TAG, and 120 CLP in Gram-positive bacteria and 10 LPG, 14 LPE, 124 PE, 37 PG, 13 TAG, and 22 CLP in Gram-negative strains. Key fatty acids, such as palmitic acid, palmitoleic acid, stearic acid, and oleic acid, were prominently featured. Univariate analysis further highlighted distinct lipidomic signatures among the bacterial strains, revealing elevated levels of phosphatidylethanolamine (PE) and phosphatidylglycerol (PG) in Gram-negative bacteria associated with DFI. In contrast, Gram-positive strains demonstrated increased or uniquely fluctuating levels of triglyceride (TAG) and cardiolipin (CLP). These findings not only underscore the utility of MALDI-TOF MS in bacterial lipidomics but also provide valuable insights into the lipidomic adaptations of bacteria in diabetic foot infections, thereby laying the groundwork for future studies aimed at constructing microbial lipid libraries for enhanced bacterial identification.

## Introduction

Diabetes is classified as a civilization disease that has a significant impact on the entire society. It is a chronic metabolic disease that results from impaired insulin secretion or action. One of the major complications of diabetes is not only hyperglycemia but also the diabetic foot syndrome (DFS).^1^ According to the World Health Organization (WHO), diabetic foot syndrome is defined as “foot ulceration associated with neuropathy and various degrees of ischemia and infections”.^2^ Diabetic foot ulcer (DFU) and diabetic foot infection (DFI) are among the most common complications of diabetic foot syndrome. DFU and DFI cause high morbidity, which is manifested by systemic toxicity, gangrene formation, and amputation of the lower limbs.^3,4^ DFI can appear as a result of a minor scratch, through scrapes, and blisters that often lead to diabetic foot ulcers. In contrast, diabetic ulcers often lead to infections.^3^ DFI is an infection of the soft tissues or bones that can lead to hospitalization and even lower limb amputation.^5,6^

A common phenomenon in chronic wounds is the presence of various species of microorganisms on their surface. Whether a wound heals or deteriorates depends on the microbiome.^7^ Due to the disturbed or impeded wound healing process in diabetics, damage to the skin on the feet is an easy target for pathogenic microorganisms and a convenient place for their development. Monitoring microorganisms from wounds plays an important role in treating diabetic foot infections and is challenging due to usually the polymicrobial nature of the infection, rapid changes in species composition occurring as the disease progresses, and coexistence of commensal bacteria as well as true and opportunistic pathogens.^8^ Both aerobic and anaerobic bacteria are involved in the pathogenesis of foot wound and ulcer infections. Among them, we can distinguish both Gram-positive bacteria (especially *Staphylococcus aureus* and beta-hemolytic streptococci) and Gram-negative bacteria (*Enterobacteriaceae* and *Pseudomonas* bacteria).^9−11^ Moreover, correlations between the intestinal microflora and bacteria with DFI have been noticed.^12,13^ Intestinal bacteria participate in digestive processes and contribute to the synthesis of nutrients beneficial for the body, support the absorption of electrolytes and minerals, play an important role in destroying toxins, prevent the development of harmful pathogens, support the immune system, and can affect inflammation. A study by Wang et al. showed a positive correlation between the intestinal microflora of mice and the concentration of lipopolysaccharides (LPS) in the plasma. LPS is a building block of the bacterial cell wall and is a component of the intestinal microflora, and thus may contribute to the inflammation of diabetes.^14^

Among the methods that have significantly contributed to the transformation of the field of microbial diagnostics are mass spectrometry platforms targeting microbial products, primarily proteins.^15,16^ Given this, pathogens identification by matrix-assisted laser desorption/ionization time-of-flight mass spectrometry (MALDI-TOF MS) analysis of high abundance proteins (ribosomal, among others) is emerging as the dominant technology in many clinical laboratories.^17^ Nevertheless, the application of such an approach meets some constraints like a commonly encountered failure to differentiate some closely related species that demonstrate high genotypic similarities (e.g., *Escherichia coli* vs *Shigella, Mycobacterium tuberculosis* complex, *Enterobacter cloacae* complex and so on) or different phenotypes (e.g., antibiotic resistant/sensitive), laborious protein extraction procedures required for microorganisms encased in the complex, thick cell walls, or poor efficiency in detecting pathogens directly in the clinical specimens.^18,19^ Due to the limitations mentioned above associated with MALDI profiling of bacterial proteins, the attention of researchers is directed to the search for new molecular targets that may allow overcoming the prevailing limitations, among which the most promising approach seems to be a lipidomics study.^20^ Lipids perform many important and unique biological functions that aim to maintain cell homeostasis. Lipids are the main structural component of cell membranes, so they influence such properties of the cell membrane as its fluidity and curvature as well as all types of interactions taking place in the membrane. Moreover, lipids are involved in the processes of energy transport and storage as well as signal transmission in the cell. Maintaining the concentration of individual lipids building cellular structures or participating in the cell signaling pathways is crucial for maintaining cellular homeostasis and is strictly regulated by lipid metabolism.^21−23^ Therefore, changes or defects in lipid metabolism have been associated with the pathogenesis of diabetes.^24^ The qualitative and quantitative composition of cell lipids may also be related to the presence of various phenotypic features of microorganisms.^25^ Therefore, the structure and functioning of the cell membrane are related to the qualitative and quantitative composition of the membrane lipids.

Microbial lipidomics has soared as a novel method of lipid analysis using MALDI-TOF MS that has the potential to address some of the challenges encountered by the proteomic approach and thereby complement them regarding the identification and classification of bacteria.^26,27^ It is known that some lipids show species-specific characteristics; therefore, they can be used as a bacteria chemical barcode during MS analysis and complement common protein-based microbial identification platforms.^28^ For example, Leung et al.^29^ found out that glycolipids can be successfully used for identification and distinguishing clinically important ESKAPE pathogens (*Enterococcus faecium*, *Staphylococcus aureus*, *Klebsiella pneumoniae*, *Acinetobacter baumannii*, *Pseudomonas aeruginosa*, and *Enterobacter* spp.) when MALDI-TOF MS analysis in negative ion mode is applied. Despite the significant progress that has been made in lipid analysis using MS, there is still a great need for their further development, including sample preparation and analysis conditions optimization (lipids are far more dependent on microbial environmental conditions than proteins) as well as bioinformatics resources for the sake of building of robust and accurate databases comparable in size and variety to the protein mass spectra libraries currently provided by commercial systems.^30,31^

The main and foremost objective of the conducted study was to accurately identify and provide detailed characterization of the lipidomic profiles of bacteria obtained from wound samples of individuals suffering from diabetic foot infections. The lipidomic analysis aimed to determine qualitative differences in the composition of cellular lipids among different strains of Gram-positive and Gram-negative bacteria. For this purpose, lipid fingerprints obtained from lipidomic analysis of lipid extracts, utilizing matrix-assisted laser desorption/ionization (MALDI) techniques and advanced chemometric techniques, were compared. It should be emphasized that there are existing studies in the scientific literature concerning lipidomic analysis conducted in individuals with diabetic foot ulcers.^32,33^ Additionally, a comparison of the lipid profiles of Gram-positive and Gram-negative bacteria was performed. The information obtained from these studies will be extremely valuable for future research endeavors focused on the construction of microbial lipid libraries for identification purposes.

## Materials and Methods

### Clinical Samples

The superficial swab samples from 15 patients from Provincial Polyclinical Hospital in Torun (Poland) from infected diabetic foot wounds were collected using a flocked swab (ESwab Collection System, Copan, Murrieta, CA, USA) by applying the Levine technique. In order to avoid contamination of the bacterial flora, the wounds were cleared of necrotic or nonviable tissue and rinsed with physiological saline before the swab was taken. After wound debridement, samples for bacterial culture were obtained by swabbing the wound (the swab was rotated over a 1 cm^2^ area of the viable non-necrotic wound tissue). The samples were immediately placed into a liquid transport medium (Amies δswab, Deltalab, Nemours, France) and transported to the Centre for Modern Interdisciplinary Technologies, where they were stored at −80 °C. The Ethical Committee approved the studies (Bioethical Commission’s permission no. 68/2019). Information about gender and age of the investigated DFI patients is provided in Table S1.

### Investigated Strains

39 bacterial isolates representing 15 different species were obtained from diabetic foot infections: *E. faecalis* (10 strains), *S. aureus* (7 strains), *C. striatum* (3 strains), *S. simulans* (2 strains), *S. dysgalactiae*, *H. kunzii*, *S. epidermidis*, and *S. pyogenes* – 1 strain each (all Gram positive bacteria), and *P. mirabilis*, *E. coli* – 3 strains each, *M. morganii*, and *K. oxytoca* – 2 strains each, *P. aeruginosa*, *P. vulgaris*, *P. cloacae* – 1 strain each (all Gram-negative bacteria) were used in research.

### Chemicals

Methanol, acetonitrile, trifluoroacetic acid (TFA), chloroform, sodium chloride, and water (all of high purity grade) were purchased from Sigma-Aldrich (Steinheim, Germany). The applied matrix 2,5-dihydroxybenzoic acid (DHB) and mass standards kit for calibration were from Sigma-Aldrich.

### Lipid Extraction from Bacteria

The extraction of the lipid fraction from bacteria was carried out according to the Folch et al. procedure with modifications.^34^ Bacterial pellets (approximately 50 mg of each DFI strain) were dissolved in 2 mL of chloroform/methanol (2:1, vol/vol) mixture and 0.5 mL of sodium chloride (0.05 M NaCl), respectively. The solutions were ultrasonicated for 10 min at room temperature, shaken for 10 min on a rotary shaker (200 rpm), and centrifuged at 5000 rpm for 20 min. After centrifugation, the lower layer was removed, and the process was repeated adding 0.5 mL of chloroform to the upper phase. The lower phases were combined and evaporated on a Labconco CentriVap DNA concentrator (Kansas City, USA). The extracted lipids were stored at −20 °C for future analysis.

### MALDI-TOF MS Analysis

Mass spectrometric measurements were performed by using a MALDI-TOF/TOF MS instrument (Bruker Daltonics, Bremen, Germany). The instrument was equipped with a modified neodymium-doped yttrium aluminum garnet (Nd: YAG) laser (1-kHz Smartbeam-II, Bruker Daltonik) operating at the wavelength of 355 nm which was used for all measurements. The extraction voltage was 25 kV, and gated matrix suppression was applied to prevent the saturation of the detector by matrix ions. All spectra were acquired in reflector positive mode within an *m*/*z* range of 200–1600 at 80% of laser power and global attenuator of 50%. All mass spectra were acquired and processed by using flexControl and flexAnalysis software, respectively (both from Bruker Daltonik).

The extracted lipids were dissolved in 0.5 mL of methanol. A 10 mg amount of DHB matrix was dissolved in 1 mL of the mixed solution (30:70, acetonitrile/0.1% TFA in water). AnchorChip MALDI target plate (anchor diameter of 800 μm; Bruker Daltonik GmbH, Bremen, Germany) was used for sample deposition. The mass spectra were calibrated by using the cesium triiodide cluster. Each sample was analyzed 3 times.

All of the lipid species were identified by using the LIPID MAPS online database (http://www.lipidmaps.org).

### Nomenclature of Lipid Species

Lipid nomenclature involves a systematic framework for identifying and characterizing lipid species. These species are assigned symbols like PL *x*:*y*, which carry crucial insights into their overall fatty acid (FA) composition. Within this system, the abbreviation “PL” indicates the lipid class, such as phosphatidylcholine or triglycerides. The value ‘*x*’ denotes the total count of carbon atoms in the lipid’s structure, while ‘*y*’ signifies the cumulative count of double bonds present in the fatty acid residues within that specific lipid species. To provide a more comprehensive breakdown of individual fatty acid constituents within the lipid structure, distinct positions are specified. The underscore notation (PL x1:y1_x2:y2) is for positions that are unspecified along the glycerol backbone. This labeling allows for finer elucidation of the lipid’s makeup.

### Statistical Analysis

The Shapiro–Wilk test was performed to investigate data distribution and therefore selected appropriate methods for comparisons between lipid means in different bacteria. The Mann–Whitney U test was applied to verify statistically relevant differences between lipid responses among isolated bacterial species. The aforementioned methods were conducted on IBM SPSS v.23 software (IBM Corp., Armonk, NY, USA). A significance criterion of *p* < 0.05 was considered. The following methods were performed in R environment, using RStudio v.1.2.1335 console (PBC, Boston, MA, USA). Obtained *p* values were adjusted for multiple comparisons using the Benjamini–Hochberg method (p.adjust function). Boxplots aiming to graphically show differences between lipid content in Gram-positive and Gram-negative bacteria were built using “ggpubr”. Network analysis employing the “sna” package was used to reveal connections between bacterial species and DFI cases—in this case, input was a binary matrix where 1 and 0 referred to species presence and absence, respectively. The package “igraph” was employed to build a lipid pathway analysis network, displaying hierarchical relationships between the detected lipids in most DFI-related bacteria. The input consisted of a list of edges linking lipid molecules to its subspecies and nodes associated with total ion intensity.

## Results and Discussion

### Lipids Profiles of Gram-Positive and Gram-Negative Bacteria

**Figure 1:**
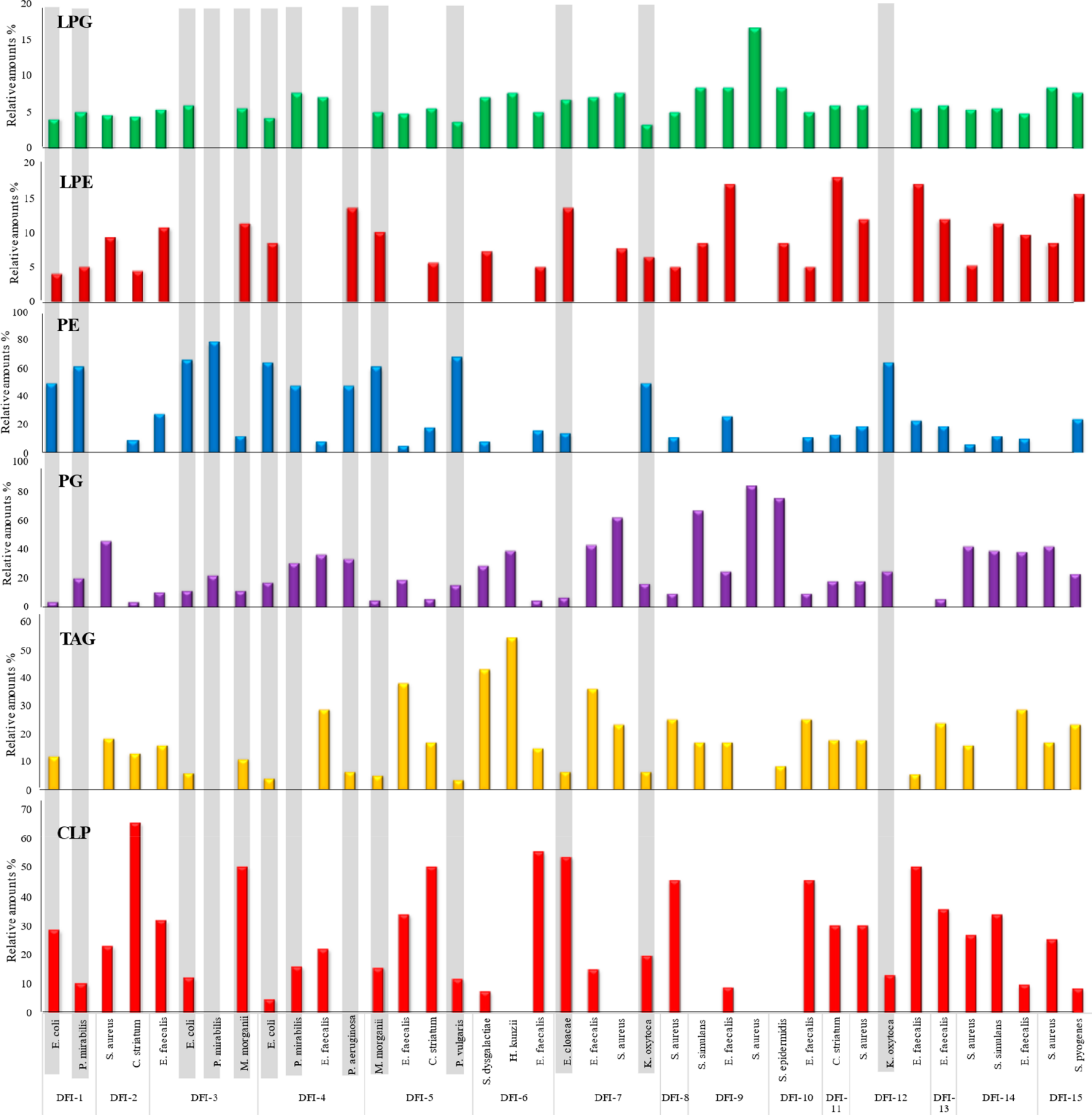
Abundance of different lipid classes in Gram positive and Gram-negative bacteria. Each color represents another lipid class: LPG – lyso-phosphatidylglycerol, LPE – lyso-phosphatidylethanolamine, PG – phosphatidylglycerols, PE – phosphatidylethanolamine, TAG – triglyceride, CLP – cardiolipin. Gray shade – Gram-negative bacteria.

The lipidome of Gram-positive and Gram-negative bacteria are mainly: phosphatidylglycerols (PGs), lysophosphatidylglycerols (Lys-PGs), phosphatidylethanolamines (PEs), lysophosphatidylethanolamines (Lys-PEs), triglycerides (TAGs) and cardiolipins (CLPs). Particular classes of lipids found in Gram-positive and Gram-negative bacterial cells extracted from people with DFI, is presented in Figure 1. These lipids are primarily building components of the cell membrane; therefore, the structure and functioning of the cell membrane are related to the qualitative and quantitative composition of membrane lipids. The balance in the lipid composition of the cell membrane also affects the proper distribution of membrane proteins and, consequently, membrane transport, DNA replication and cell division.^35^ Changes in the permeability of the cell membrane resulting from the modification of its structure may be related to the reduced penetration of antibiotics into the cell.^36^

The study used 39 bacterial isolates, which were obtained from superficial swab from 15 patients; 8 species were Gram-positive and 7 were Gram-negative bacteria. Figure 1 shows relative amounts of all of the identified obtained lipids from Gram-positive and Gram-negative bacteria. As a result of the studies (Figure 1), a large variability in the qualitative composition of lipids was observed among Gram-positive and Gram-negative bacteria. In Gram-positive bacteria: 26 LPGs, 33 LPEs, 43 PEs, 114 PGs, 89 TAGs and 120 CLPs molecules of lipids were identified, and in Gram-negative bacteria, 10 LPGs, 14 LPEs, 124 PEs, 37 PGs, 13 TAGs and 22 CLPs molecules of lipids were identified, respectively (Table S2). Differences in the lipid profile patterns between Gram-positive and–negative bacteria have been previously demonstrated in the literature. Zhang et al.^31^ using desorption electrospray ionization mass spectrometry (DESI-MS) to measure lipids directly from 16 different bacterial species pointed out that it is easy to differentiate Gram-positive and Gram-negative bacteria since spectra of Gram-negative bacteria were more abundant in fatty acids, LPG, LPE, PE, and PG, while spectra of Gram-positive bacteria contain lipopeptides and very limited abundance of PG, PE, and LPG. Similar findings noted Lellman and Cramer^32^ using the liquid atmospheric pressure (AP) MALDI MS technique to lipid profiling of multiple bacterial strains including *E. coli*, *Klebsiella pneumoniae*, *Campylobacter jejuni*, *S. aureus*, *S. epidermidis*, *S. pyogenes*, *Lactobacillus brevis*, *E. faecalis*, *Enterococcus hirae*, and *P. aeruginosa* where PEs and PGs were more abundant among Gram-negative species while cardiolipins and lyso-phospholipids were dominant signals within MS spectra of Gram-positive ones. Results of our studies proved that Gram-negative bacterial cells consist of a much higher number of PEs compared to the Gram-positive ones, which makes PEs a potentially good biomarker for the indication of a Gram type of bacteria present in the sample. Nevertheless, the revealed much higher number of LPGs and LPEs in the Gram-positive bacteria is inconsistent with the results of the above-mentioned works as well as with the statement that Gram-positive bacteria demonstrated much lower phospholipid signals resulting from the structural characteristics of their cell membrane. One possible explanation of such phenomena may be related to differences in the process of the sample preparation and ionization techniques since Zhang et al.^31^ used ESI techniques while in the case of a second cited work^32^ authors used atmospheric pressure conditions as well as a simple ethanol/formic acid extraction protocol commonly used for proteins detection and liquid support matrix (LSM), formed of matrix chromophore molecules and the addition of a viscous support liquid such as glycerol which use resulted in the production of ‘electrospray ionization-like’ multiply charged ions. Indeed, Lellman and Cramer revealed great differences in lipids MS profiles between liquid and solid AP-MALDI-MS conditions.^32^ It is known that the analysis of lipid mixtures is challenging for many reasons. Different classes of lipids affect the intensity of others, so MALDI mass spectra can change if they are recorded at different analyte concentrations. Moreover, the choice of the extraction protocol may also significantly influence the result of the lipid profiling since techniques based on the use of chloroform favor detection bulk lipids such as PEs, while more polar compounds like lysophosphatidic acid or (poly)phosphoinositides are incompletely extracted. Furthermore, some lipids may stick to the precipitated proteins present in the form of the white ring at the interface between the aqueous and the organic phase during lipids extraction via the Folch method and therefore may be lost since only the organic layer is used for further analysis. Matrix selection also matters since for example DHB, which is the most commonly used matrix in lipid analysis, tends to ionize phospholipids with quaternary ammonia groups such as lyso(monoacyl)-phosphatidylcholine or sphingomyelin, whereas negatively charged phosphatidylinositol and phosphatidylserine are detected with very low sensitivity when the positive ion detection mode is used.^33^ All together this may explain the differences in the lipidomic profiles of the Gram-positive and -negative species revealed in our and cited works and emphasize the need for looking for optimal and standardized lipid extractions and analysis protocols to facilitate interlaboratory trials comparison.

In addition, variability in the lipid composition within one group of strains was also observed. For example, the *E. coli* strain that was identified in patient DFI-1 (1-LPG, 1-LPE, 12-PE, 1-PG, 3-TAG, and 7-CLP), DFI-3 (1-LPG, 11- PE, 2-PG, 1-TAG, and 2-CLP), and DFI-4 (1-LPG, 2-LPE, 15-PE, 4-PG, 1-TAG, and 1-CLP) has different lipid compositions. The highest number of phosphatidylethanolamines lipid molecules (18 molecules of PE) was identified for the *P. vulgaris* strain in the DFI-5 patient, 10 phosphatidylglycerols for the *S. aureus* strain in the DFI-2 patient, 8 triglycerides for the *E. feacalis* strain in the DFI-5 patient, and 15 cardiolipin for *C. striatum* in patient DFI-2, respectively. PG molecules have been identified in every bacterium, except *E. faecalis* (DFI-12).

On the example of *E. coli* (−) and *S. aureus* (+), a large difference in the lipid composition of bacterial cytoplasmic membranes can be observed. Major lipids of *E. coli* (DFI-1) glycerol phosphate based lipids include zwitterionic phosphatidylethanolamine (PE, 48% of membrane), anionic phosphatidylglycerol (PG, 4%), cardiolipin (CLP, 28%) and triglycerides (TAG, 12%). In contrast, in *S. aureus* (DFI-2), PE is 0%, PG is 45%, and CLP is 23%; the remaining 14% are zwitterionic lipids: lyso-phosphatidylglycerol 5% and lyso-phosphatidylethanolamine 9%. The observed differences in lipid content may reflect differences in lipid metabolism and membrane structure between the strains studied. The cell wall of Gram-positive bacteria is thick due to the combination of murein with another polysaccharide polymer—teichoic acid or glycerol polymer, which is a component of phosphatidylglycerol. Murein can also combine with existing proteins and create a multilayer structure itself.^40^ In Gram-negative bacteria, there is an additional layer of the outer membrane.^41^ The outer membrane is composed of proteins and lipopolysaccharide (LPS).^42^ Lipopolysaccharide consists of lipid A, a core part, and a polysaccharide chain (referred to as the O antigen). Lipid A has a sugar core, which consists of two molecules of glucosamine. Then, arabinose is attached to one end of the disaccharide and phosphate residues and ethanolamine (which is part of phosphatidylethanolamine) to the other.

### Lipid-Profiling by MALDI-TOF for Microbial Identification

**Figure 2:**
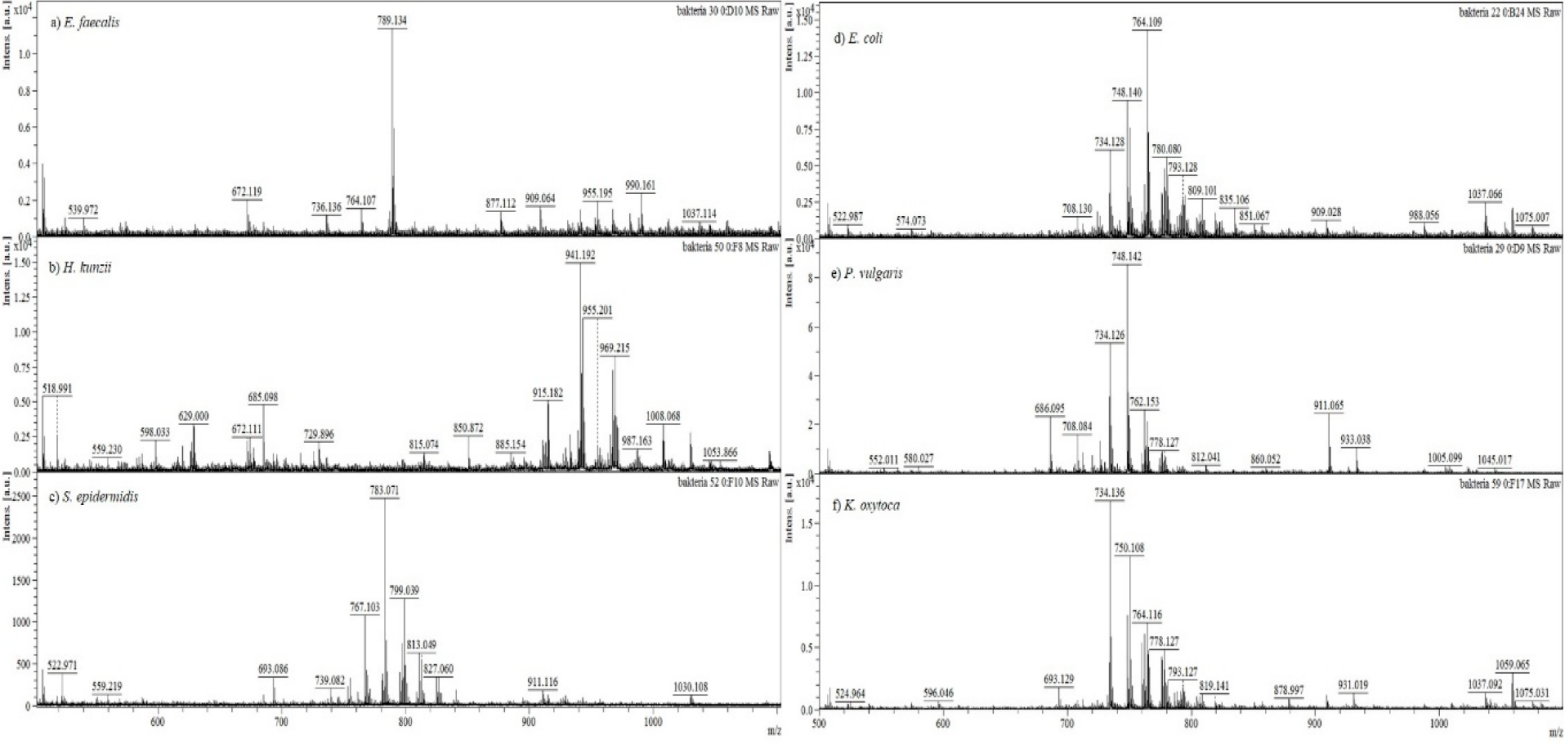
MALDI-TOF MS mass spectra recorded in positive mode of Gram-positive: (a) *E. faecalis*; (b) *H. kunzii*; (c) *S. epidermidis*, and Gram negative bacteria: (d) *E. coli*; (e) *P. vulgaris*; (f) *K. oxytoca*.

The lipid profile of Gram-positive and Gram-negative bacteria was recorded using MALDI-TOF MS. The scanning range was set to *m*/*z* 400–2000. Figure 2 shows mass spectra detected in positive ionization for selected bacterial strains. It can be seen from the spectrum that the dominant ions detected are mostly intact lipids distributed in the *m*/*z* range of 600–1000. This is consistent with previous work by Lellman and Cramer, where the largest distribution of lipid ion signals for Gram-negative species was detected in the *m*/*z* 650–800 range (mainly associated with an abundance of PEs), and that for Gram-positives in the *m*/*z* 900–1000 range was due to the presence of LPG and cardiolipins.^38^ In addition, it was observed that the lipid signals detected in Gram-negative bacteria were much stronger than those in Gram-positive bacteria. Probably, the thick layer of peptidoglycan in Gram-positive bacteria may hinder the partial release of lipids from the bacterial cell membranes.^43^

108 combinations of fatty acids in lipid molecules were identified for bacteria isolated from the diabetic foot infection (Table S2). The identified acids contained from 12 to 22 carbon atoms in the acyl chain and from 0 to 6 double bonds. The most common fatty acids for all lipid molecule-building DFI bacterial strains are C16:0 (palmitic acid), C16:1 (palmitoleic acid), C18:0 (stearic acid), and C18:1 (oleic acid). Omega-3 (ω-3) and omega-6 (ω-6) fatty acids such as C18:2 ω-6 (linoleic acid), C18:3 ω-3 (α-linoleic acid), C20:4 ω-6 (arachidonic acid), and C22:6 ω-3 (docosahexaenoic acid) were also identified in the tested bacterial strains (Table S2). The content of particular classes of lipids, as well as the type of fatty acids constituting the side chains of lipids (number of carbon atoms, degree of unsaturation, presence or absence of branching) in DFI bacterial cells, depends on many factors, e.g. growth phase of bacterial cells and culture conditions.^44^

*Escherichia coli* was for many years a model organism in the description of cell membrane lipids.^44^ Bacterial proteins are the subject of intensive research, and the state of knowledge about the role of phospholipids in bacterial strains is scarce. The arrangement and composition of membrane lipids and modifications of the “polar heads” of phospholipids affect the interactions between other molecules and the environment. The structure and dynamics of plasma membrane phospholipids play an important role in the penetration of antibiotics into the cell.^45^ Therefore, an important goal of the research was to determine the importance of lipid structures in bacteria isolated from DFI. In the conducted studies, the lipid profile was characterized including fatty acids that build specific molecules of phospholipids and triglycerides. Lipid molecules species such as PE 12:0_12:0, PE 16:1_16:1, PG 16:1_15:0, PG 16:0_16:1, PE 17:0_17:0 have only been identified for *P. vulgaris* (DFI-5) bacteria. It can be considered that the phospholipids identified as differentiators in this study may be potential biomarkers of DFI in the case of *P. vulgaris* bacteria. In addition, all identified lipids are major components of the cell membranes. PG 15:1_15:0 was only identified for *H. kunzii* (DFI-6), TAG 18:0_18:0_20:2 for *S. dysgalactiae* (DFI-6), and PE 18:2_18:3, PE 20:1_20:1, TAG 16:1_18:3_18:3 for *P. aeruginosa* (DFI-4), respectively. It was also observed that for the *E. coli* strain that was isolated from patients 1 and 3, two different potential biomarkers TAG 20:2_20:2_21:2 and PG 16:0_15:0 were identified, respectively. Differences in the lipid composition within the same strain result from the high phenotypic variability of the bacteria and variable fat metabolism. Many studies have shown that temperature, pressure, pH, and nutrients determine the level of fats, including the composition of fatty acids and lipid groups found in bacterial membranes.^44,46^ Differences in the composition of fatty acids contained in lipids are their important chemotaxonomic features and may be of practical importance in the diagnosis of this group of bacteria at the species level. A similar correlation was observed for the *C. striatum* strain isolated in patients 2 and 5. These two species differed in the composition of the fatty acids in the cardiolipin class: CLP 16:0_18:1_16:1_17:0, CLP 16:1_18:1_17:0_18:0, CLP 16:1_18:1_18:0_18:0, CLP 22:0_22:0_22:0_21:1 (DFI-2) and CLP 18:0_18:0_18:0_18:0, CLP 18:1_19:1_18:1_19:1, CLP 18:1_19:1_18:1_19:0, and CLP 20:1_21:0_21:0_21:0 (DFI-5). Combinations of fatty acids in cardiolipin molecules such as CLP 16:1_16:1_16:1_16:1, CLP 18:1_19:0_18:1_19:0, and CLP 20:1_20:1_21:0_21:0, CLP 20:1_20:0_21:0_21:0 occur only in the strain: *P. mirabilis* (DFI-4), *S. aureus* (DFI-15), and *E. faecalis* (DFI-3), respectively. The dominant lipid molecules for all bacterial strains were LPG 12:0 (92%), PE 18:2_20:4 (69%), and CLP 18:1_19:0_19:1_19:0 (72%).

As a result of the conducted research, it was observed that the lipid profile for each bacterial strain is unique and nonrepeatable. The analysis of fatty acids in lipid molecules reveals diverse lipid composition. The classes of lipids in the cell membranes of Gram-positive and Gram-negative bacteria differ. Moreover, the identified lipids are recognized as components of cell membranes. Lipid diversity also arises from disruptions in phospholipid and triglyceride metabolism during the infection process, leading to cellular dysfunction and death. All identified and characterized lipid molecules serve as differentiators and can be regarded as potential biomarkers for DFI. Noticed observations appeared to prove the concept by Solntceva et al. that MALDI analysis of the lipids can provide subspecies-level classification supporting microbial identification at a species level yielded by protein fingerprinting, thus helping accomplish several diagnostic goals simultaneously.^20^ Furthermore, recognition of microbial species-specific lipids could be a game changer in rapid microbial diagnostics since the detection of the distinct microbial lipids directly from body fluids could allow avoiding the need for culture on agar plates or in liquid medium providing that a suitable method of their enrichment from the samples is given.^17^ Depending on the length of the acyl chain and the degree of unsaturation, fatty acids in lipid molecules can have either a positive or negative effect on systemic insulin sensitivity.^47^ It is likely that a different insulin sensitivity is the cause of changes in fatty acid composition. Clinical studies have demonstrated that reduced concentrations of polyunsaturated fatty acids (PUFA) in phospholipids lead to decreased insulin sensitivity.^48^ Additionally, abnormal fatty acid metabolism results in decreased glucose uptake, leading to excessive intramuscular triglyceride (IMTG) accumulation and insulin resistance.^49^

Obtained results prove the concept that lipid signals obtained during MALDI analysis are well suited to act as a chemical fingerprint for differentiating microorganisms.^20^ The significant advantage of the proposed MALDI approach is that information about fatty acids composition is also provided; however, without the need of using a multistep protocol required when gas chromatography (the most traditional and old technique) is applied, which involves saponification of bacterial cells in sodium hydroxide, methylation with hydrochloric acid, lipid extraction, and a wash at a basic pH which typically takes several hours.^50^ The powerful impact of the MALDI approach in the differentiation of the bacteria based on their lipidomic profiles, even at the strain level, has been recently proven for *E. coli* strains isolated from DFI wounds that differed in beta-lactams antibiotic resistance level.^51^

### Statistical Evaluation of Obtained Lipid Profiles

**Figure 3:**
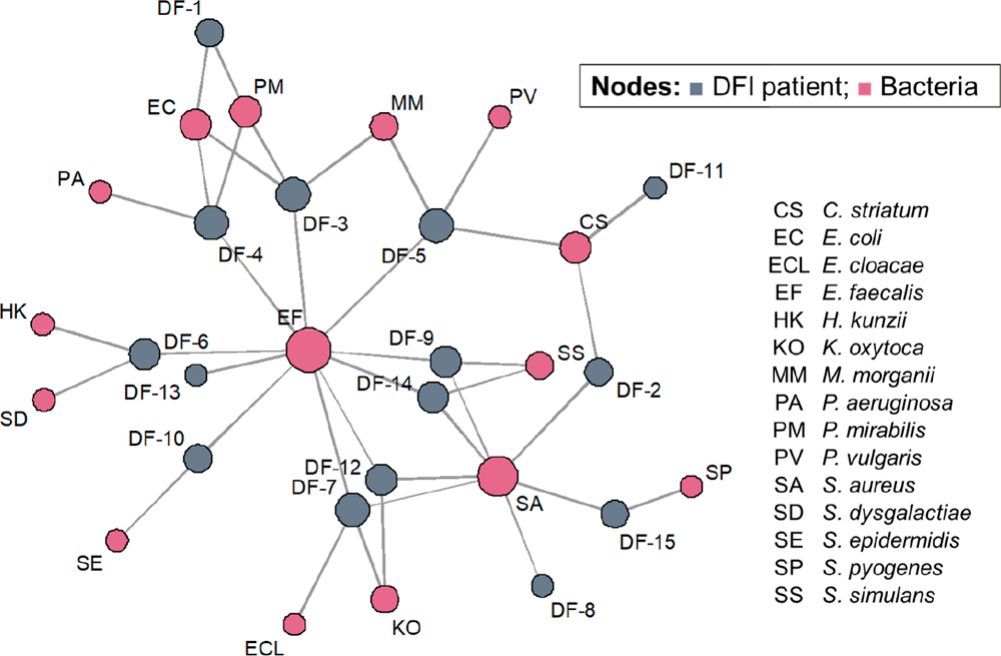
Network analysis showing the relationships between DFI samples and identified bacteria species. DF – diabetic foot infection case.

The Figure 3 is a network showing the interrelations between DFI cases and detected bacteria, giving a graphical representation of the bacterial prevalence in DFI samples. The most recurrent simplified microbiome in DFI consisted of *E. faecalis* coexisting with *S. aureus*. The bacterial variety ranged from 2 to 4 species coexisting in the same sample site—each of these configurations were present in equal proportions, representing 80% of sampled DFI, whereas colonization by only one species was less frequent (20% of DFI cases). From assessed bacterial mixtures, only one of them was composed exclusively of Gram-negative bacteria, with most of them consisting of the homogeneous presence of Gram-positive bacteria (40% of the cases). Mixtures containing both Gram-negative and Gram-positive species represented 33.4% of DFI samples. Such a trend is confirmed by other bacteriological studies on DFI, which report that Gram positive *cocci* are the most frequently isolated species.^52^

**Figure 4:**
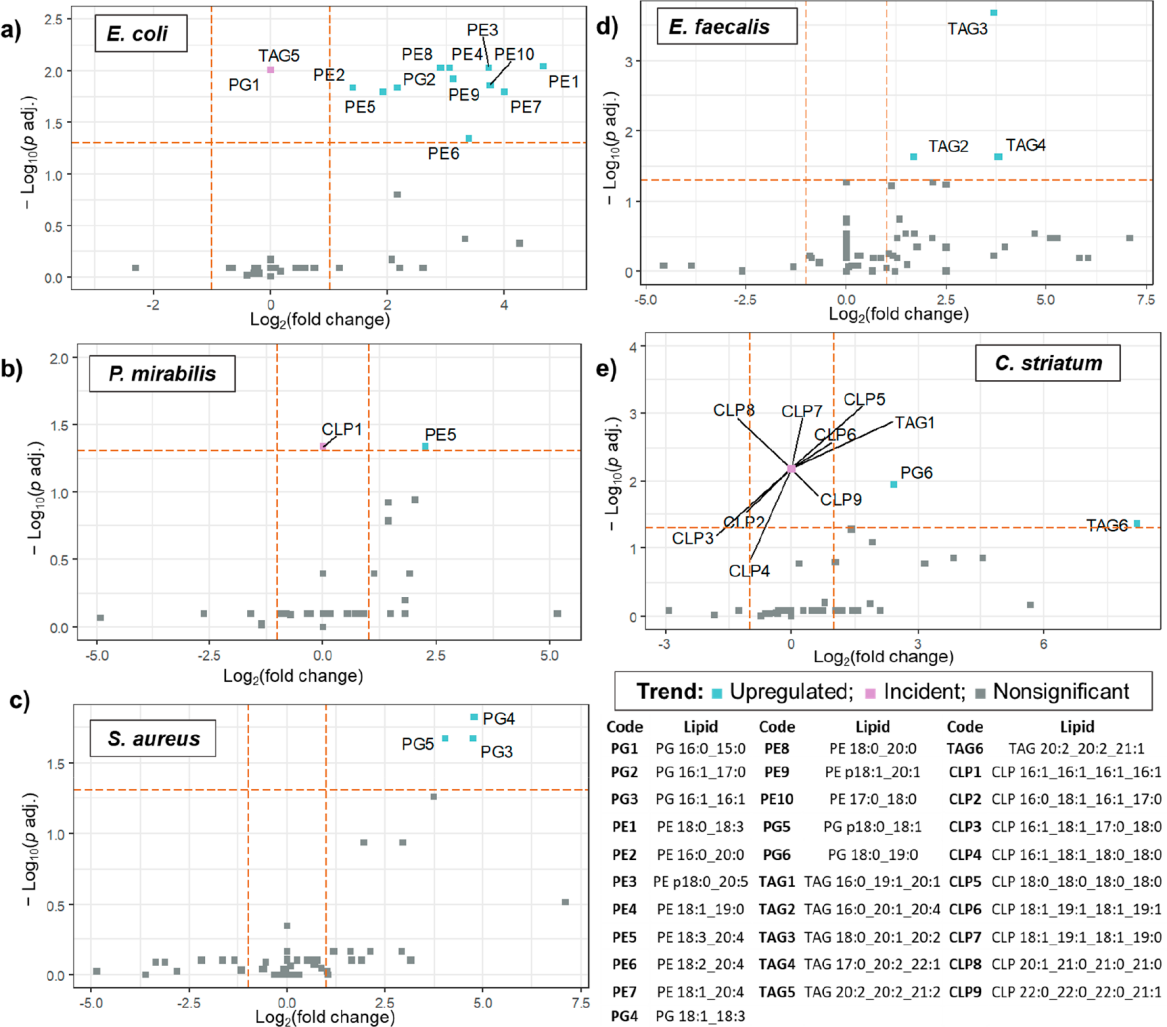
Feature relevance plot, showing discriminant lipids found for (a) *E. coli*, (b) *P. mirabilis*, (c) *S. aureus*, (d) *E. faecalis*, and (e) *C. striatum*. The horizontal threshold line shows where *p* adjusted <0.05. PG – phosphatidylglycerols, PE – phosphatidylethanolamine, TAG – triglyceride, CLP – cardiolipin.

Among the bacterial species identified in DFI samples, those with a higher incidence were *E. faecalis* (10, 25.6%), *S. aureus* (7, 17.9%), *E. coli* (3, 7.7%), *C. striatum* (3, 7.7%), and *P. mirabilis* (3, 7.7%). First, lipids characteristic for each of the most predominant DFI bacteria were investigated. For this purpose, a statistical comparison of lipid composition was performed considering a single species against all others. The Figure 4 shows a features plot of the obtained statistical significance (expressed as the negative common logarithm of adjusted *p* value) versus the magnitude of the change (expressed as the binary logarithm of the fold-change in relation to lipid intensities in other species). In this manner, lipids plotted toward the top part of the graph displayed a greater relevance (lower *p* values). Lipids plotted at the right part of the graph presented an increased response in relation to the remaining bacteria (blue dots), and those located across the *y*-axis were incident only in the given bacteria (purple dots). Since these are the features significantly altered in comparison to the whole set of bacterial strains isolated from DFI samples, they can serve as potential biomarkers of the given bacterial species. Such lipid molecules may be further considered in predictive models for reliable bacterial identification based on lipidomics.

This approach showed that *E. coli* species (Figure 4a) were mainly characterized by a greater concentration of several PE variants as well as distinguished incidences of TAG 20:2_20:2_21:2 and PG 16:0_15:0. Another Gram-negative bacteria, *P. mirabilis* (Figure 4b), also displayed increased levels of a PE derivate (PE 18:3_20:4). Besides that, CLP 16:1_16:1_16:1_16:1 was differentially present in their lipid composition. In the case of *S. aureus* (Figure 4c), these species were distinguished by the augmented levels of PG. Conversely, *E. faecalis* (Figure 4d) displayed remarkably increased concentrations of TAG variants in the lipid content. Finally, the lipid composition of *C. striatum* (Figure 4e) was mainly marked by the presence of unique CLP species. Additionally, for these bacteria, other differentiated lipids comprised elevated TAG derivates (TAG 16:0_19:1_20:1—exclusively incident in these species—and TAG 20:2_20:2_21:1), as well as PG 18:0_19:0.

Indeed, PE is reported as the most abundant lipid in *E. coli*, accounting for 70–80% of total membrane lipids. In these bacterial species, PE is particularly necessary for the functioning of lactose permease—a membrane associated protein responsible for the transport of β-galactosides into the cell, for which PE deficiency results in unsuccessful coupling of substrate uptake. Moreover, PE also plays an important part in *E. coli* adhesion, possibly due to its influence on lipopolysaccharide biosynthesis.^53,54^ Discriminating elevated levels of a PE variant in *P. mirabilis* is also expected, as PE content is generally superior in Gram-negative bacteria when compared to Gram-positive species.^55^ Studies have shown that in actively growing *S. aureus*, PG is the most predominant phospholipid, which agrees with the present findings. During the stationary phase, PG content tends to decrease as CLP is augmented. PG to CLP in *S. aureus* membranes also appears to be induced by phagocytosis promoted by human neutrophils. Such alterations in phospholipid metabolism are probably coordinated by the regulation of CLP synthase.^56^ A few TAG species were elevated only in *E. faecalis*. Such lipids were also detected during the exponential growth phase of these bacterial species by previous research. The role of TAG lipids in bacteria is still not extensively studied. TAGs mainly serve as a reservoir of fatty acids, also contributing for the regulation of membrane fluidity and working as an electron sink.^57^ TAG accumulation tends to occur for some bacterial species when the cells develop in nonrelated carbon sources, like glucose.^58^ In addition, a differential lipid profile has been observed for daptomycin-resistant *E. faecalis*, marked by reduced levels of PG and LPG in comparison to agent-sensitive strains. This indicates an increased consumption of PG substrate toward Glycerophospho-Diglucosyl-Diacylglycerol biosynthesis in resistant *E. faecalis*, which can serve as an intermediate for the *de novo* production of TAG.^57^ Finally, specific CLP species were found only in *C. striatum*. A previous investigation on extracts of *Corynebacterium glutamicum* reported that after PG, CLP species were the most abundant species. However, a higher CLP content was found tightly associated with the peptidoglycan–arabinogalactan complex of the bacterial cell wall.^59^ Such a lipid configuration of *Corynebacterium* species may explain why the CLP variety was the most discriminating aspect of DFI-derived *C. striatum*.

**Figure 5:**
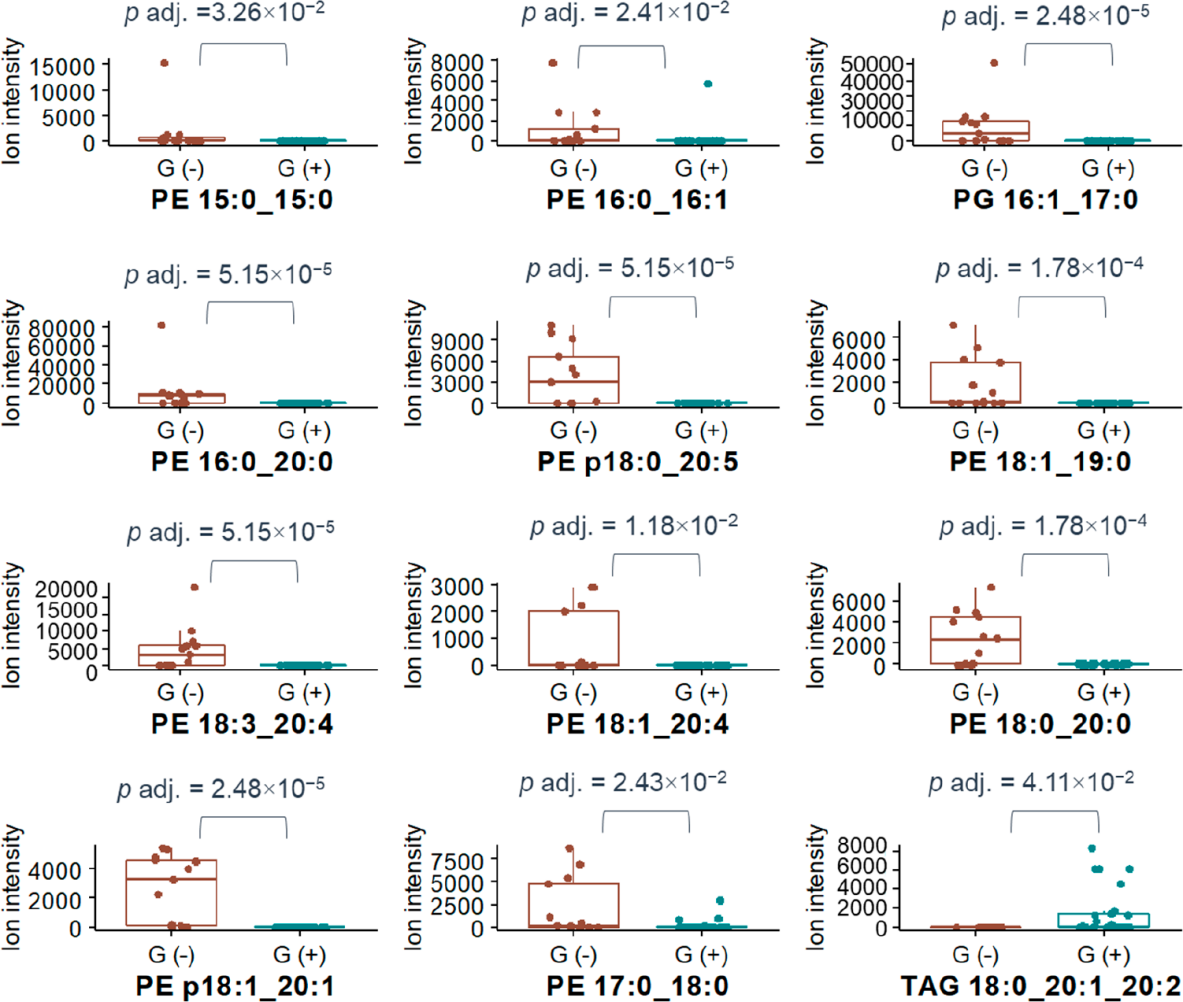
Statistically significant changes in lipid intensities for Gram-negative and Gram-positive bacteria. PG – phosphatidylglycerols, PE – phosphatidylethanolamine, TAG – triglyceride.

Figure 5 presents boxplots showing the lipids that displayed the greatest statistically relevant differences between their responses in Gram-positive and Gram-negative bacteria. As expected, due to their major lipid composition, Gram-negative bacteria were described by a significantly higher content of several PE species. PE 16:1_17:0 and PE p18:1_20:1 were the most unique lipid species (presenting the lowest *p* values), differentiated between the two bacteria groups. For this reason, such species can be considered as molecular indicators, characteristic for Gram-negative bacteria. On the other hand, TAG 18:0_20:1_20:2 displayed significantly greater responses for Gram-positive bacteria.

### Lipid Pathway Considerations Regarding DFI Bacteria

**Figure 6:**
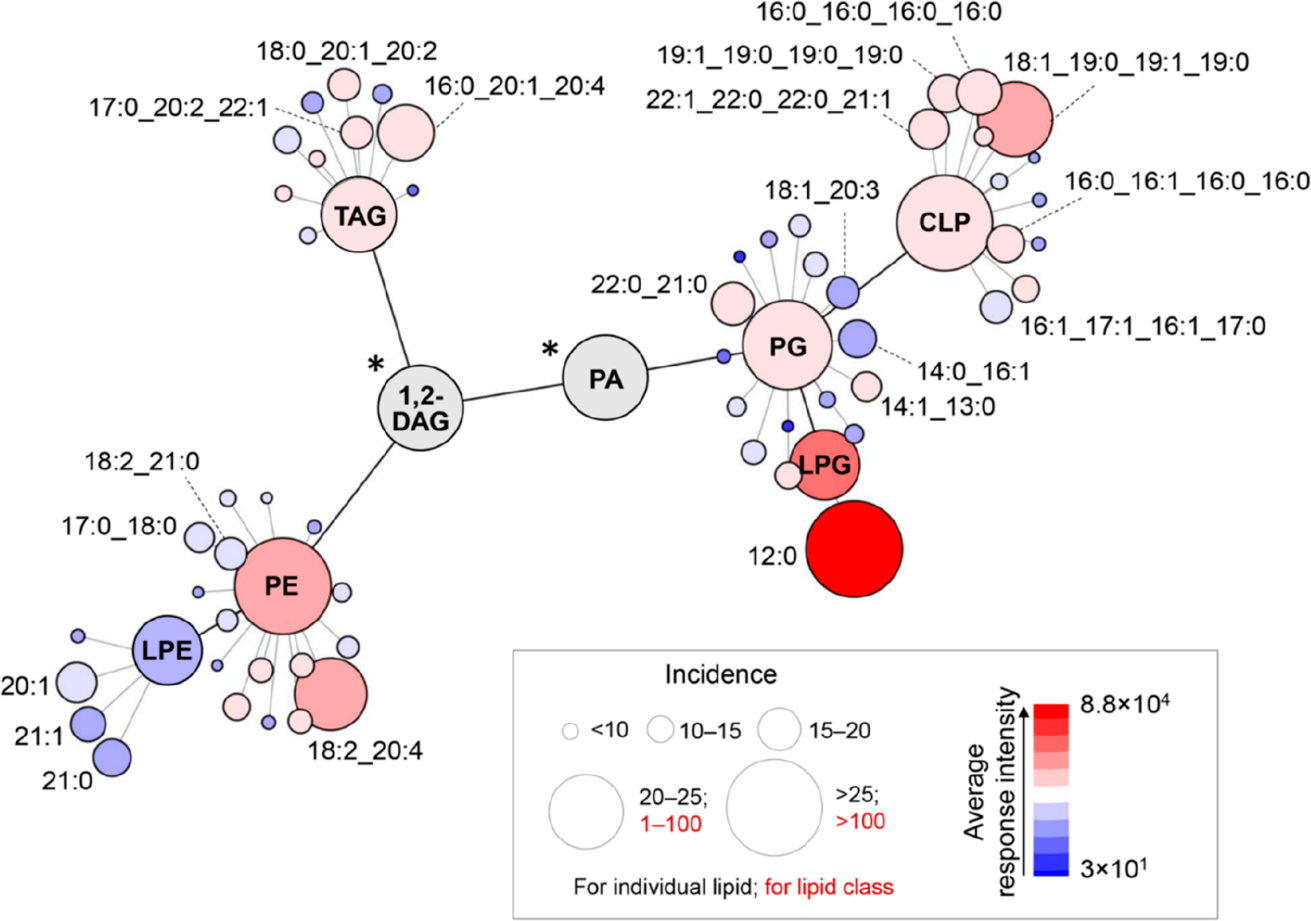
Lipid pathway network based on the DFI-bacteria lipidome. LPG – lyso-phosphatidylglycerol, LPE – lyso-phosphatidylethanolamine, PG – phosphatidylglycerols, PE – phosphatidylethanolamine, TAG – triglyceride, CLP – cardiolipin. * – Not measured metabolite: 1,2-DAG – 1,2-diacylglycerol, PA – phosphatidic acid.

A representation of the configuration of main lipid pathways in DFI-related bacteria is shown in Figure 6. In this, only lipids incident more than 10 times in DFI-lipidome are depicted. The connections in black show the link between lipid classes, while connections in gray refer to the relation between the individual lipids and their main classes. The size of the nodes is proportional to the prevalence of detected lipids (how many times they were detected within the samples), whereas the color code refers to the average intensity of the lipid ion. This approach clearly shows that PG, CLP, and PE are the main lipid classes identified in the total lipidome of DFI bacteria. Among them, some specific variants displayed consistently high responses. It is the case of LPG 12:0. LPG is a commonly referred membrane lipid in several Gram-positive species, however scarcely discussed regarding Gram-negative bacteria.^60^ In the present study, LPG 12:0 was detected with considerably high intensity, also for Gram-negative species. Other studies indicate that this lipid class is potentially involved in bacterial resistance mechanisms. Daptomycin-resistant strains of *S. aureus* often display an augmented LPG membrane content together with an enhanced surface positive charge. Such a lipid configuration possibly stabilizes membrane integrity, avoiding structural defects and an increased vulnerability to cationic antimicrobial agents.^61^ Low pH-inducible genes (*lpiA*) have been detected in Gram-negative α-proteobacteria like *Rhizobium tropici*, encoding homologues of MprF—the protein gene responsible for LPG synthesis in *S. aureus*. Deletion of *lpiA* turned *R. tropici* susceptible to cationic antimicrobial peptides, revealing that LPG confers increased resistance in acidic growth conditions.^60^ Therefore, LPG detection also among Gram-negative DFI bacteria can suggest such an adaptation mechanism in the species isolated in the present study. Among CLP, as previously mentioned, CLP 18:1_19:0_19:0 was the most preponderant variant. It has been hypothesized that an increased cardiolipin content may contribute to bacterial resistance. Molecular dynamics simulations have showed that negative membrane curvature promoted by CLP can contribute to cell protection against antimicrobial peptides, by counteracting their proneness to induce a positive curvature in target membranes.^62^ Moreover, previous research demonstrated that clinical *S. aureus* strains performed metabolic adaptation. In this case were observed point mutations in the phospholipid biosynthesis gene (cls2) which encodes the synthesis of CLP, leading to an alteration in anionic membrane phospholipid composition. The described configuration resulted in a phenotype characterized by increased membrane CLP and reduced PG.^63^ Indeed, PG variants displayed intensities much lower than those of CLP variants in the present study. PG is referred to as a potential chemoattractant, stimulating the recruitment of neutrophils to the infection site.^55^ For this reason, a shift toward increased synthesis of LPG and CLP, leading to reduced PG levels, may indicate a greater resistance of DFI-bacteria.

PE was the lipid more frequently detected among bacterial species, presented also in greater amounts of total lipid content, with the PE 18:2_20:4 variant configured as the most common PE molecule observed among DFI-bacteria. It appears that only a small fraction of PE is converted into LPE once such species are detected with lower intensities. As discussed previously, PE may play an important role in bacterial adhesion to substrates, enabling efficient colonization. Additionally, other experiments with PE-deficient *E. coli* have demonstrated possible functions in cell division and stress response activation.^54^

The study of bacterial membrane lipids has long been recognized as a critical avenue for understanding bacterial physiology, stress adaptation, and host–pathogen interactions. For instance, alterations in bacterial lipid profiles have been implicated in various physiological processes, such as cell shape maintenance, envelope integrity, and adaptability to environmental stress. These lipidomic changes are not merely structural but functional and serve as potential targets for novel antimicrobials. This traditional understanding of bacterial lipidomics is now being significantly enriched by the advent of advanced analytical techniques, among which MALDI stands out as a complementary and versatile tool.^64^

In the current landscape of lipidomics, MALDI serves as a robust adjunct to other established methods like RPLC-MS. Its utility extends from clinical settings, as demonstrated by Buszewska-Forajta et al.^65^ in their analysis of lipids in prostate tissue, to cardiovascular research where Moerman et al.^66^ employed it for lipid analysis in carotid atherosclerotic plaques. Beyond its role as a complementary technique, MALDI has shown its mettle in identifying a wide array of lipid classes across diverse biological systems. For example, Walczak-Skierska et al.^67^ employed MALDI-TOF/MS for lipidomic analysis of lactic acid bacteria strains, while Bhandari et al.^68^ used it for lipid profiling in insect models. These applications underscore MALDI’s versatility and its growing role in lipidomic research, a point further emphasized by Nikitina et al.,^69^ who described lipid profile changes in induced pluripotent stem cells.

Moreover, MALDI’s applicability is not confined to academic research, but extends to practical and clinical settings. M. Hajjar et al.^70^ utilized MALDI TOF in negative-ion MALDI-TOF for the analysis of LPS or lipid A preparations to identify *Francisella tularensis* strains. In the food industry, the technique has been employed for evaluating dietary supplements containing live bacteria, as shown by Lorbeg et al.^71^ In plant research, Wang et al.^72^ used it for studying lipid distribution in peanut seeds. Importantly, in the realm of microbial identification, particularly for closely related or drug-resistant strains, MALDI’s role is being increasingly recognized. This is corroborated by emerging trends in the development of lipid-based databases for microbial identification, as demonstrated by the work of Maria-Theresia Gekenidis et al.^73^ and P. Lasch et al.,^74^ who have shown that MALDI-TOF MS can achieve deeper taxonomic identification and is particularly useful in clinical settings. Our approach of employing MALDI for building lipid libraries is not an isolated endeavor but is well-aligned with current scientific advancements. It offers a promising avenue for bacterial identification, particularly when considering the growing interest in lipid-based databases and the technique’s broad applicability across various research and clinical settings.

The network presented by Figure 6 may also depict an enrichment pattern useful for spotting a microbiome associated with DFI through a lipidomics approach. In order to confirm the usefulness of such lipid enrichment profile in identifying a phenotype derived from DFI bacteria, further studies would be necessary to compare paired bacterial species colonizing non-DFI wounds. Recent studies showed that 2 h of incubation in the presence of antibiotic (cefotaxime in that case) is enough to detect distinctive lipid patterns of beta-lactam-resistant and -sensitive *E. coli* strains.^51^

## Conclusions

An analytical methodology has been developed that enables lipid analysis of Gram-positive and Gram-negative bacteria in people with diabetic foot disease. This approach makes it possible to understand the lipid composition of the cells of these bacteria and determine the lipidomic differences among strains with different phenotypes. The proposed procedure includes sample preparation and lipid identification using the MALDI-TOF MS technique. In addition, the use of a high-resolution TOF mass spectrometer and the created lipid database of such bacteria as *E. faecalis*, *S. aureus*, *C. striatum*, *S. simulans*, *S. dysgalactiae*, *H. kunzii*, *S. epidermidis*, *S. pyogenes*, *P. mirabilis*, *E. coli*, *M. morganii*, *K. oxytoca*, *P. aeruginosa*, *P. vulgaris*, *P. cloacae* enables the identification and proposition of the structures of the main cell lipids of these bacteria: phosphatidylglycerols, lyso-phosphatidylglycerols, phosphatidylethanolamines, lyso-phosphatidylethanolamines, triglycerides, and cardiolipins. The use of the MS/MS technique also enables determination of the length of the acyl substituents in the detected lipids.

By combining the MALDI-TOF MS technique with biostatistical and chemometric data analysis, the developed methodology can be successfully applied to lipidomics studies in various biological contexts—e.g. building lipid reference libraries for species identification, selection potential microbial-specific biomarkers for direct detection in clinical specimens, and subspecies differentiation. In addition, large variability in the qualitative composition of lipids within one group of strains was observed, which indicates a high phenotypic variability of bacterial cells and the need to analyze lipid extracts from many strains, cultured in several replicates, in order to extract information on lipidomic differences between bacterial strains. The observed differences in lipid content may reflect differences in lipid metabolism and membrane structure between the tested strains of Gram-positive and Gram-negative bacteria.

The achievements of the presented work indicate the high potential of the MALDI technique as a relatively simple and rapid method of classifying bacteria based on lipidomic profiles and respond to the recent appeal of the research community that, so far, routine identification of microorganisms by MALDI-TOF MS based on the identity and abundance of lipids has not been explored extensively to differentiate species and deserves more investigation.^20^

## Supporting Information Available

Table S1: Details information about gender and age of the investigated DFI patients (PDF)
Table S2: All identified combinations of fatty acids in lipid molecules species (XLSX)
