# Supplementary material for: Lipidomics Characterization of the Microbiome in People with Diabetic Foot Infection Using MALDI-TOF MS
Source: Anal Chem. 2023 Oct 25;95(44):16251–62. doi: 10.1021/acs.analchem.3c03071 (PMC10633811; doi:10.1021/acs.analchem.3c03071)
Supplement: Supplementary file 1 — ac3c03071_si_001.pdf [file ac3c03071_si_001.pdf]

Lipidomics characterization of the microbiome in people with diabetic foot infection using MALDI-TOF MS

Justyna Walczak-Skierska[1,2*], Fernanda Monedeiro[1], Ewelina Maślak[1], Michał Złoch[1,2]

[1]Centre for Modern Interdisciplinary Technologies, Nicolaus Copernicus University in Toruń, Wileńska 4 Str., 87-100 Toruń, Poland

[2]Chair of Environmental Chemistry and Bioanalytics, Faculty of Chemistry, Nicolaus a Copernicus University in Toruń, Gagarina 7 Str., 87-100 Toruń, Poland

** Correspondence: walczak-skierska@umk.pl*

Table S1. List of investigated DFI patients with information about gender and age.

Patient	Gender	Age
P1	Male	80 yrs.
P2	Female	63 yrs.
P3	Female	85 yrs.
P4	Female	87 yrs.
P5	Male	74 yrs.
P6	Male	57 yrs.
P7	Male	73 yrs.
P8	Female	68 yrs.
P9	Male	62 yrs.
P10	Male	54 yrs.
P11	Male	53 yrs.
P12	Male	81 yrs.
P13	Female	65 yrs.
P14	Male	71 yrs.
P15	Male	65 yrs.
